# Lupus nephritis in Bangladesh: unfolding the story

**Published:** 2016-01-02

**Authors:** Muhammed Mubarak

**Affiliations:** Department of Histopathology, Sindh Institute of Urology and Transplantation (SIUT), Karachi, Pakistan

**Keywords:** Lupus nephritis, ISN/RPS classification, Systemic lupus erythematosus

**Implication for health policy/practice/research/medical education:**

Lupus nephritis (LN) represents the most dreadful complication of patients with systemic lupus erythematosus (SLE). It is responsible for a major share of morbidity and mortality in these patients. The rate of development of this complication varies widely. The pathological lesions of LN are nowadays classified according to International Society of Nephrology/Renal Pathology Society (ISN/RPS) classification of 2003. This classification has been validated in many clinicopathological studies and has good inter-observer reproducibility. LN can involve any of the four components of the kidney parenchyma in variable combinations and at different time points in the disease course. Glomerular lesions represent the most commonly involved component and form the mainstay of pathological classifications of LN.

Lupus nephritis (LN) represents the most dreadful complication of patients with systemic lupus erythematosus (SLE). It is responsible for a major share of morbidity and mortality in these patients. The rate of development of this complication varies widely (1,2). The reported figures vary from 40% to 80%. LN can involve any of the four components of the kidney parenchyma in variable combinations and at different time points in the disease course. Glomerular lesions represent the most commonly involved component and form the mainstay of pathological classifications of LN (3).

Renal biopsy plays an important role in the diagnosis, classification, treatment and prognostication of patients with LN. The pathological lesions of LN are nowadays classified according to International Society of Nephrology/Renal Pathology Society (ISN/RPS) classification of 2003 (4). This classification has been validated in many clinicopathological studies and has good inter-observer reproducibility. Clinicopathological studies have helped immensely in defining the full spectrum of clinical and laboratory features of patients with SLE who develop LN. These studies have also highlighted racial and geographic differences in the spectrum of LN patients. These studies help in identifying the local demographics of the patients with this disease and help clinicians in the management of these patients (1-6).

Baqui et al in the current issue of this journal have analyzed a cohort of 34 patients with biopsy proven LN (7). This is a retrospective study and the number of cases is quite small. These may be considered limitations of the study. In addition, the cross-sectional nature of the study also limits its value. There is no information about the treatment of the disease. These are some of the major limitations of the studies from this region. The follow-up of patients is often erratic. There are issues with the compliance to treatment. The authors also included both pediatric and adult patients in the study. Nevertheless, it is a welcome addition to the existing meager literature on this condition in Banglandesh. Such studies can be considered as the beginning of hypothesis-based prospective studies and should be encouraged, especially in third world countries. These authors found arthralgia and edema as the most common clinical features of LN in this study population. Almost two-thirds of the patients belonged to LN class IV, which is the most severe form of LN. Among the clinical and laboratory features, arthralgia and serum creatinine showed significant correlation with LN pathological classes (7).

The authors also noted some important differences from other regional studies. For example, the mean age of patients in their series was a decade lower, as compared with some other countries, such as, Singapore and China (5,6). This led the authors to suggest that the onset of LN in Bangladesh is earlier as compared with some other races, where LN is common. Similarly, the gender distribution of the cases also showed divergent results as compared with a study from Singapore, again pointing towards racial differences (5). Regarding pathological classes of LN, it was observed that class IV LN was the single most predominant class in this study. This is concordant with many other studies on this subject and reflects to a major extent the biopsy policies in LN patients (1-6,8-11). Obviously, the patients with more severe involvement undergo biopsies more often than those who are less severely affected. When the authors correlated clinical and laboratory features with LN classification, it was found that only arthralgia and serum creatinine correlated with pathological classes of LN (7). These results are also contradictory to what some previous studies have reported. The authors were not able to explain the cause of this discrepancy, nor was it the objective of this study.

In conclusion, the authors of the present study deserve compliments on sharing their experience of LN patients from a country, where very little is known about the clinicopathological spectrum of the disease. We hope that the authors will continue with this research work and will initiate hypothesis-based prospective studies to answer the questions raised by this study.

## Author’s contribution

MM was the single author of the manuscript.

## Conflicts of interest

The author declared no competing interests.

## Ethical considerations

Ethical issues (including plagiarism, misconduct, data fabrication, falsification, double publication or submission, redundancy) have been completely observed by author.

## Funding/Support

None.
